# New anti-HCV agents in the pipeline

**DOI:** 10.1186/1471-2334-14-S2-S15

**Published:** 2014-05-23

**Authors:** Fabien Zoulim

**Affiliations:** 1 INSERM U1052, Hospices Civils de Lyon, University of Lyon 1, Lyon, 69000, France

## 

Opportunities to treat infection with hepatitis C virus (HCV) are evolving rapidly. From the introduction of interferonα monotherapy in the early 90s' to the approval of telaprevir and boceprevir based triple therapies with pegylated interferonα and ribavirin in 2011, the chances of curing patients infected with HCV genotype 1 have improved dramatically to reach approximately 70%. Significant further improvements, which may cure virtually all hepatitis C patients with an all oral, interferon-free regimen will become available in the very near future. These new direct acting antivirals (DAA) target the viral polymerase with either nucleoside analogues or non nucleosidic inhibitors, the viral protease, and the viral NS5A protein. Several clinical trials have now shown that a combination of sofosbuvir (nucleosidic polymerase inhibitor) with daclatasvir or ledipasvir (NS5A inhibitors), or sofosbuvir with simeprevir (protease inhibitor), or a combination of ABT-450 (proease inhibitor) with ritonavir (ABT-450/r), the nonnucleoside polymerase inhibitor ABT-333 and the NS5A inhibitor ABT-267, can achieve sustained virologic response in up to 95% of naive patients or previously treated patients, even in patients who failed prior treatment with first generation protease inhibitor. As these DAAs are becoming available in early access treatment programs, treatment strategy studies are being performed to optimize treatment regimens with respect to the choice of DAAs and treatment duration, based on viral genotypes, prior treatment exposure, and the presence of liver cirrhosis. The next challenge will be to identify HCV infected patients in the general population and to facilitate access to treatment.

